# The Poplar Rust-Induced Secreted Protein (RISP) Inhibits the Growth of the Leaf Rust Pathogen *Melampsora larici-populina* and Triggers Cell Culture Alkalinisation

**DOI:** 10.3389/fpls.2016.00097

**Published:** 2016-02-17

**Authors:** Benjamin Petre, Arnaud Hecker, Hugo Germain, Pascale Tsan, Jan Sklenar, Gervais Pelletier, Armand Séguin, Sébastien Duplessis, Nicolas Rouhier

**Affiliations:** ^1^Institut National de la Recherche Agronomique, Centre INRA Nancy Lorraine, UMR 1136 Interactions Arbres/MicroorganismesChampenoux, France; ^2^Faculté des Sciences et Technologies, UMR 1136 Interactions Arbres/Microorganismes, Université de LorraineVandoeuvre-lès-Nancy, France; ^3^The Sainsbury LaboratoryNorwich, UK; ^4^Groupe de Recherche en Biologie Végétale, Université du Québec à Trois-Rivières, Trois-RivièresQC, Canada; ^5^CRM^2^, Equipe BioMod, Faculté des Sciences et Technologies, UMR 7036, Université de LorraineVandoeuvre-lès-Nancy, France; ^6^CNRS, CRM^2^, Equipe BioMod, Faculté des Sciences et Technologies, UMR 7036Vandoeuvre-lès-Nancy, France; ^7^Natural Resources Canada, Canadian Forest Service, Laurentian Forestry Centre, QuébecQC, Canada

**Keywords:** antifungal activity, peptide elicitor, plant immunity, *Populus trichocarpa*, rust fungus, obligate biotroph

## Abstract

Plant cells secrete a wide range of proteins in extracellular spaces in response to pathogen attack. The poplar rust-induced secreted protein (RISP) is a small cationic protein of unknown function that was identified as the most induced gene in poplar leaves during immune responses to the leaf rust pathogen *Melampsora larici-populina*, an obligate biotrophic parasite. Here, we combined *in planta* and *in vitro* molecular biology approaches to tackle the function of RISP. Using a RISP-mCherry fusion transiently expressed in *Nicotiana benthamiana* leaves, we demonstrated that RISP is secreted into the apoplast. A recombinant RISP specifically binds to *M. larici-populina* urediniospores and inhibits their germination. It also arrests the growth of the fungus *in vitro* and on poplar leaves. Interestingly, RISP also triggers poplar cell culture alkalinisation and is cleaved at the C-terminus by a plant-encoded mechanism. Altogether our results indicate that RISP is an antifungal protein that has the ability to trigger cellular responses.

## Introduction

Plant cells respond to changes in their environment by secreting peptides in their extracellular space (Krause et al., 2013; Matsubayashi, 2014; Simon and Dresselhaus, 2015). Understanding how these peptides function is key to understanding plant responses. Secreted peptides belong to two main categories: the antimicrobial peptides (AMPs) that directly interfere with invaders, and the peptide hormones that function as signal molecules which coordinate further responses.

Antimicrobial peptides constitute a major component of the inducible plant immune system (Dodds and Rathjen, 2010). Their gene expression is tightly controlled and usually induced upon pathogen infection (Tao et al., 2003). AMPs exert a direct toxic effect against microbial parasites, often through the disruption of their cell integrity (van Loon et al., 2006; Poon et al., 2014). The antimicrobial activities of these peptides are commonly characterized using *in vitro* assays which consist in applying purified peptides to microbial cultures and then in evaluating growth reduction (Vasconcelos et al., 2011; Poon et al., 2014). At the structural level, plant AMPs typically present rigid 3D structures whereas many AMPs from other eukaryotes are intrinsically disordered in aqueous solutions (Zasloff, 2002; Haney et al., 2009; Pelegrini et al., 2011).

Peptide hormones (also known as peptide elicitors) are signaling molecules involved in cell-to-cell communication for coordinating plant development or stress responses (Butenko et al., 2009; Fuglsang et al., 2014; Tabata and Sawa, 2014; Bartels and Boller, 2015). These are usually small peptides, with a size comprised between 11 and 50 amino acids, and which are often released by the cleavage of larger protein precursors (or pro-proteins). They function in the apoplast where they bind to cell-surface receptors to trigger cellular responses (Monaghan and Zipfel, 2012; Krause et al., 2013; Haruta et al., 2014). Cell culture alkalinisation assays, during which extracellular pH shifts toward basic values, are used as proxies to detect plant cellular responses to peptides (Yamaguchi and Huffaker, 2011). Complementary assays measuring ROS levels, MAP kinase activation, gene expression or levels of plant hormones are often used to determine the exact nature of the responses (activation of immune responses for example).

Poplar trees are used in agroforestry for wood production, but their susceptibility to the leaf rust fungus *Melampsora larici-populina*, an obligate biotrophic pathogen, causes severe losses in plantations (Duplessis et al., 2009). Since the release of the poplar (*Populus trichocarpa*) genome sequence, many research efforts have been devoted to the identification of components of the poplar immune system that govern resistance to *M. larici-populina* (Duplessis et al., 2009; Hacquard et al., 2011). Notably, Rinaldi et al. (2007) reported that the *Rust-induced secreted protein* (*RISP*) gene is highly induced in poplar leaves challenged by an avirulent isolate of *M. larici-populina*. RISP is a small (82 amino acids), cationic (pI 9.5), cysteine-rich (4) protein predicted to be secreted in the apoplast. These observations prompted the authors to hypothesize that RISP is a component of the poplar immune system, possibly working as an antifungal agent. More recently, *RISP* gene position in the *P. trichocarpa* genome was refined and it was found at close vicinity of a leucine-rich repeat receptor-like protein (LRR-RLP) gene (Petre et al., 2014). Interestingly, both genes present similar promoter regions and expression profiles in response to *M. larici-populina* infection. Considering that LRR-RLPs are cell-surface receptors, we recently suggested that RISP could also be a peptide hormone or a precursor of such peptide (Petre et al., 2014).

In the present study, we have tested whether RISP (i) is a secreted protein, (ii) is an AMP that can inhibit the growth of *M. larici-populina*, and (iii) is a peptide hormone able to trigger cellular responses in poplar. We assessed the first point by determining the subcellular localisation of a RISP-mCherry fusion transiently expressed in *Nicotiana benthamiana*. After producing a recombinant RISP and investigating its biochemical and structural properties, we addressed the second question by examining RISP interaction with *M. larici-populina* urediniospores and evaluating its ability to inhibit fungal growth. In order to test the third hypothesis, we measured the capacity of RISP to promote poplar cell culture alkalinisation.

## Materials and Methods

### Biological Material, Growth Conditions, and Inoculation Procedures

Hybrid poplar cultivar ‘Beaupré’ (*Populus trichocarpa* x *Populus deltoides*) and *M. larici-populina* isolates 98AG31 (pathotype 3-4-7) and 93ID6 (pathotype 3-4), were grown and used as previously detailed (Rinaldi et al., 2007). *Laccaria bicolor* strain S238N was maintained at 25°C in dark conditions on Pachlewsky P5 medium (Di Battista et al., 1996), with transfer to fresh media every 4 weeks. *Magnaporthe oryzae* strain Guy11 was maintained at 25°C in the dark on complete medium (Talbot et al., 1993), with transfer to fresh media every 2 weeks. For longer storage, mycelium fragments were conserved on sterile Whatman paper at –20°C. For spore production, a frozen-stock was inoculated on complete medium. Spores were retrieved after 12 days by raking the mycelium in sterile water and purified by filtration on sterile Joseph paper, washed twice in 50 mL sterile water by gentle centrifugation and immediately used.

### Sequence Analyses and Bioinformatic Procedures

Sequences were retrieved from the *P. trichocarpa* ‘Nisqually-1’ genome sequence hosted on the Phytozome portal (version 2.2, currently v3 assembly^1^). Sequence alignments were performed using the online programs Multalin^2^ and ClustalW^3^. Intrinsically disordered regions were predicted using Globplot online^4^. Protein signal peptides were predicted on the SignalP 3.0 server^5^ and protein parameters were calculated with the Protparam program^6^. Disulfide bonds were predicted using the diANNA 1.1 Web server^7^.

### RNA Isolation and RT-qPCR Analyses

Time-course infection of poplar leaves by either virulent or avirulent isolates of *M. larici-populina* as well as water-agar (mock-inoculated treatment), RNA isolation and cDNA synthesis were performed previously (Petre et al., 2011). A pair of primers (forward: GCGGTAGTAGCAAACAAAGT; reverse: CTCCTAAGCACGTATACAAC) was designed to specifically amplify RISP transcripts (GenBank accession number: XP_006379231, Phytozome ID Potri.T160900.1 in *P. trichocarpa* v3.0, POPTR_0580s00210.1 in v2.2). We experimentally determined the efficiency of this primer pair to be 1.89, i.e., 89%. Reverse transcription-quantitative polymerase chain reaction (RT-qPCR) experiments were carried out as previously described (Hacquard et al., 2010), and included two biological and two technical replicates. For each biological replicate, the average value of the two technical replicates was used, provided that the two values differed by less than one Ct. Transcript expression was normalized to a reference ubiquitin transcript as previously reported (Rinaldi et al., 2007).

### Cloning and Transient Expression of a RISP-mCherry Fusion in *N. benthamiana* Leaf Cells

The open reading frame (ORF) encoding the full-length RISP (i.e., with the signal peptide) was amplified by polymerase chain reaction (PCR) from Beaupré leaf cDNA and cloned into the golden gate level 0 vector pICSL01005 (AATG/TTCG compatible overhangs^8^). The DNA fragment was then assembled with an ORF coding the mCherry (pICSL50004, TTCG/GCTT compatible overhangs) into the golden gate level 1 binary vector pICH86988 (AATG/GCTT compatible overhangs) in order to create a RISP-mCherry fusion expressed under the control of a 35S promoter (see Supplementary Table S1). The transient transformation of *N. benthamiana* leaf cells by *Agrobacterium tumefaciens* was performed as previously described (Win et al., 2011). Leaves were collected 2 days post-infiltration and pavement cells were observed with a Leica DM6000B/TCS SP5 microscope (Leica Microsystems, Bucks, UK). The mCherry was excited at 561 nm. mCherry and chlorophyll fluorescent signals were retrieved between 580–620 nm and 680–710 nm, respectively. Plasmolysis was induced with 1 M NaCl. Image analysis was performed with Fiji^9^.

### Cloning, Expression, and Purification of Recombinant Proteins

The ORF sequence encoding mature RISP (devoid of the first 23 amino acids) was amplified from *P. trichocarpa* cDNAs and cloned into pET3d and pET15b (Novagen) for producing a protein without tag or with an N-terminal His-tag respectively. For generating the RISP-GFP-His fusion, the mature RISP sequence was cloned into pCK S65T (Menand et al., 1998) for a C-terminal in-frame fusion with GFP S65T, and then subcloned into pET28a to insert a His-tag at the C-terminus of the GFP. Primers, plasmid construction and corresponding expressed proteins are presented in Supplementary Table S1. His-tagged or untagged recombinant RISP were produced in *Escherichia coli* strains BL21(DE3) pSBET or Rosetta2(DE3) pLysS, purified following a procedure previously described (Couturier et al., 2014) and dialyzed against 30 mM Tris-HCl, 1 mM EDTA pH 8.0 (TE) buffer. As a final purification step, recombinant RISP proteins were eventually incubated 10 min at 95°C to remove residual *E. coli* contaminants that precipitated upon heating. Protein concentrations were measured by spectrophotometry using a molar extinction coefficient at 280 nm of 1,640 M^–1^ cm^–1^. All the experiments involving recombinant RISP were performed with freshly prepared proteins because they precipitated upon freezing. Other recombinant proteins (His-AtBolA2, AtGrxC1, poplar His-Trxh1 and *Neisseria meningitidis* Grx) were produced and purified as previously described (Rouhier and Jacquot, 2003; Rouhier et al., 2007; Couturier et al., 2014). These proteins, used as negative controls in the antifungal and cleavage assays, were selected due to their similarity to RISP in terms of size, stability, and production/purification procedure. For ^15^N-labeled RISP production, bacteria were grown in M9 minimal synthetic medium containing ^15^NH_4_Cl (1 g/L) as previously described (Bouillac et al., 2004).

### *In Vitro* Antifungal Assays

To assess the ability of recombinant proteins to inhibit the germination of *M. larici-populina* urediniospores, 15,000 urediniospores stored at –80°C (isolate 98AG31) were resuspended in 1 mL of sterile water under vigorous agitation for 1 h, then put in contact with protein solution (the protein concentration in the tube is indicated everywhere relevant) and spread on water-agar (20 g/L) in Petri dishes. After 5 h of incubation (constant light 25 μmol/s/m^2^, 21°C), urediniospores were observed under a binocular and the percentage of germination was calculated on a minimum of 300 urediniospores for each replicate. To assess the ability of recombinant proteins to inhibit the elongation of *M. larici-populina* germ tubes, urediniospores were spread on water-agar (20 g/L) and were allowed to germinate for 4 h. Then, protein solutions were gently deposited on agar surface to avoid germ tube breaking and Petri dishes were incubated for 4 more hours. Pictures were taken after 4 and 8 h under a binocular microscope and germinating tube length was measured using the AnalySIS^B^ software (Olympus, Tokyo, Japan). To assess the ability of recombinant proteins to inhibit the mycelium growth of *L. bicolor* and *M. oryzae in vitro*, 10 μL of 100 μM recombinant RISP or control solutions were placed at the margins of growing mycelium colonies on paper disks or directly onto the medium and growth was recorded after 3 and 7 days for *M. oryzae* and *L. bicolor*, respectively. Phenotype of the hyphae was estimated by light microscopy. Alternatively, 2 mm^3^ fungal explants were transferred on appropriate fresh media and 100 μL of 100 μM recombinant RISP or control solutions were directly deposited on the explants. Growth of the fungi was recorded after 8 days. To assess the ability of recombinant proteins to inhibit the germination of *M. oryzae* spores, the latter were allowed to germinate in glass cupules with 100 μM recombinant RISP or control solutions. Germination was recorded after 6 and 24 h under a light microscope.

### Antifungal Assay on Poplar Leaves

Beaupré leaves were inoculated with a mix of *M. larici-populina* (isolate 98AG31, virulent on Beaupré) urediniospores (100,000 spores/mL) and 100 μM recombinant proteins in TE buffer, TE buffer alone or double-distilled water (ddHOH). Inoculation was performed on entire leaves as previously described (Rinaldi et al., 2007) or with a pipette on 5 cm^2^ leaf disks. Pictures were taken 7–10 days post-inoculation (dpi) unless otherwise stated. For pre-treatments by recombinant RISP prior to inoculation, 1 mL of 100 μM recombinant protein or controls were deposited with a pipette on 3 cm^2^ areas of Beaupré leaves and then inoculated with *M. larici-populina* urediniospores (100,000 spores/mL, isolate 98AG31) immediately, or 3 or 5 days later. Orange uredinia pustules were tracked from 8 up to 20 dpi.

### Recombinant Protein Reduction and Thiol Titration

Recombinant proteins were reduced with a 100-fold excess of dithiothreitol (DTT, 10 mM for 100 μM recombinant proteins) for 1 h at room temperature. DTT was removed by desalting on G25 columns (GE Healthcare, Little Chalfont, UK). For thiol titration, 20 μM of untreated and pre-reduced recombinant proteins prepared in a TE or TE-SDS 1% buffer were reacted with 100 μM 5,5′-dithiobis-2-nitrobenzoic acid (DTNB) for 1 h in the dark. Absorbance was measured at 412 nm and free-thiol content was calculated using a TNB- molar extinction coefficient of 13,600 M^–1^ cm^–1^ as previously reported (Rouhier et al., 2004).

### Structural Analyses

For CD analyses, His-RISP was adjusted to 50–200 μM in a 10 mM phosphate buffer pH 7.0, and spectra were recorded on a CD6 spectropolarimeter (HORIBA Jobin Yvon, Longjumeau, France) in triplicates at 20°C with 0.2 nm wavelength increments from 190 to 250 nm, using a 0.1 cm path length cuvette. The spectra were corrected from the buffer baseline. CD spectra of the reduced proteins were performed with 10 mM DTT added 10 min prior to analyses. For experiments with trifluoroethanol (TFE), 50 or 80% TFE was added to recombinant proteins 1 h prior to analyses. Secondary structure abundance was determined after deconvolution of the spectra with Selcon3 and ContinII softwares.

Nuclear magnetic resonance (NMR) spectra of His-RISP were obtained on a 600 MHz Bruker Avance III spectrometer equipped with a TCI cryoprobe. COSY, TOCSY (mixing time of 60 ms) and NOESY (mixing time of 150 and 300 ms) experiments were recorded on a 1 mM His-RISP sample in a 4:1 (v/v) mixture of deuterated trifluoroethanol (TFE-d3) and 5 mM phosphate buffer at pH 7.4 and 25°C. Spectra were processed in Topspin3.0 (Bruker) and referenced to TFE residual signal at 3.88 ppm.

One hundred and sixty-five distance restraints were derived from NOESY experiments. These restraints were applied in an iterative structure calculation and a simulated annealing protocol with water refinement using ARIA2.3 (Rieping et al., 2007) and CNS1.21 (Brünger et al., 1998; Brünger, 2007). Eighty structures were generated and the 20 structures of lowest energy were kept. The global backbone RMSD among the 20 lowest-energy structures is 0.63 and 1.62 Å for the 1–9 and 10–15 segments, respectively.

### Poplar Cell Suspension Alkalinisation Assay

Poplar cell suspensions (*P. trichocarpa* x *P. deltoides*; H11-11 hybrid) were maintained in Murashige and Skoog medium (pH 5.6, adjusted with KOH) by weekly transferring 10 mL of cells to 30 mL of fresh media. Cells were grown in dark conditions, at 20°C and under shaking at 160 rpm. For the alkalinisation assay, cells were used 4 days after transfer. The day before each alkalinisation assay, 5 mL of cells were aliquoted in sterile opened 50 mL centrifuge tubes and allowed to equilibrate overnight at room temperature at 160 rpm in the dark. Prior to the alkalinisation assay, cultures equilibrated 2 additional hours under room-light conditions. A constant volume of 20 μL of recombinant proteins or TE buffer adjusted to various concentrations was added and pH variations were recorded thereafter. TE buffer treatment remained stable at a pH of 4.2 during the experiment and was used to calculate the pH ratio for each time-point.

### Leaf Protein Isolation and Western Blotting Analyses

Frozen leaves were ground with a mortar and a pestle in liquid nitrogen. The powder was resuspended into the following extraction buffer [50 mM Tris-HCl pH 8.0, 1 mM phenylmethanesulfonylfluoride (PMSF), 50 mM β-mercaptoethanol] and incubated for 20 min under shaking at 4°C. After centrifugation (15 min at 30,000 *g*), the supernatant (i.e., soluble proteins) was precipitated overnight at –20°C into 80% cold acetone whereas the pellet was resuspended into another extraction buffer (50 mM Tris-HCl pH 8.0, 1% SDS) to recover insoluble proteins. After 20 min incubation and a similar centrifugation, the supernatant containing denatured insoluble proteins was also precipitated overnight at –20°C into 80% cold acetone. Proteins were recovered by centrifugation (10 min at 35,000 *g*), the pellet was resupended into 50 μL SDS 0.2% and the proteins were quantified using a BCA (bicinchoninic acid) assay. The protein concentration was adjusted to 2 μg/μL in a Laemmli buffer, denatured 10 min at 95°C and stored at –20°C. For total protein extraction, leaf powder was directly resuspended into a denaturing extraction buffer (7 M urea, 2 M thiourea, 4% [m/v] CHAPS, 0.4% [v/v] triton X-100, 10 mM DTT) and incubated for 1 h under shaking at 4°C. After centrifugation (15 min at 30,000 *g*), the supernatant corresponding to total proteins was precipitated overnight at –20°C into 80% cold acetone. Proteins were retrieved, quantified and adjusted into Laemmli buffer as specified above.

Western blotting analyses were performed as previously described (Pégeot et al., 2014). Polyclonal antibodies raised in rabbit against recombinant His-RISP were purified from the immune serum by affinity chromatography as described previously, using a CnBr sepharose bound untagged RISP (Rouhier et al., 2001). Pre-immune and depleted sera were used as controls to ensure the specificity of the signal.

### Time-Course Cleavage of Recombinant Proteins by Poplar Soluble Protein Extracts

Five percent (v/v) of purified RISP, His-RISP, or poplar His-Trxh1 were incubated with soluble protein extracts from poplar leaves for up to 24 h at room temperature under gentle shaking and with 1 mM PMSF to avoid rapid and unspecific protein degradation. The incubation was arrested by denaturing and reducing the samples (i.e., by adding Laemmli buffer and boiling the samples for 10 min at 95°C). Protein integrity was followed by western blot analyses as described above.

### Pull-Down of RISP with *M. larici-populina* Urediniospores

Urediniospores from the *M. larici-populina* (isolate 98AG31) were resuspended in sterile ddHOH with 0.05% Tween20 under vigorous agitation for 30 min. Five hundred micrograms of resuspended urediniospores were incubated with 50 μg of recombinant proteins for 5 min under gentle agitation in ddHOH. Urediniospores were washed twice with 1 mL of ddHOH by centrifugation at 13,000 *g* for 30 s. Finally, urediniospores were incubated in 100 μL of Laemmli buffer, incubated 10 min at 95°C and centrifuged again (the supernatant being the final elution). The presence of recombinant proteins in the different fractions was estimated by 15% SDS-PAGE and Coomassie blue staining. Recombinant proteins that were obtained following similar procedures used for RISP were used as negative controls.

## Results

### A RISP-mCherry Fusion Accumulates in the Apoplast in *N. benthamiana*

**FIGURE 1:**
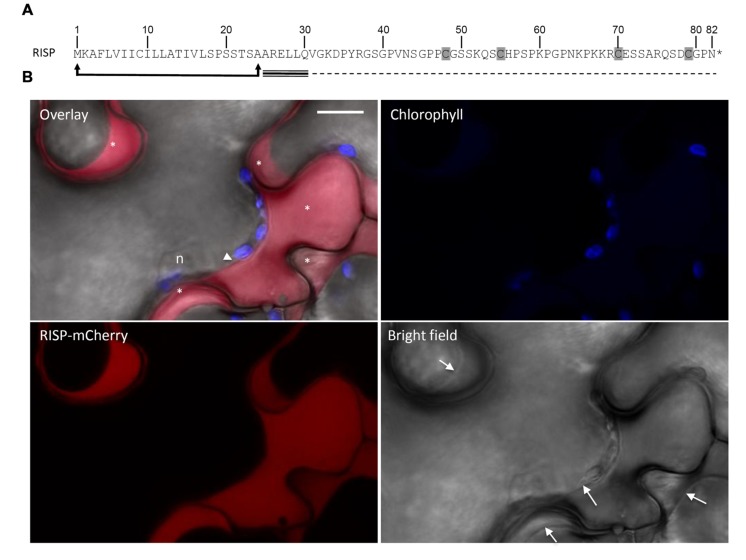
**Rust-induced secreted protein (RISP) is secreted into the apoplast. (A)** RISP amino acid sequence. The predicted secretory peptide is delimited by arrows and the four cysteines are shaded. The predicted intrinsically disordered region and the predicted α-helix are indicated with dotted and triple lines respectively. **(B)** A RISP-mCherry fusion was transiently expressed into *Nicotiana benthamiana* leaf pavement cells by the agro-infiltration method. Cells were plasmolysed with 1 M NaCl 5 min before observation. The mCherry signal was observed by live-cell imaging 2 days post-infiltration. Excitation: 561 nm. Signal collection: mCherry (red, 580–620 nm); chlorophyll (blue, 680–710 nm). Scale bar: 10 μM. n: nucleus. The white arrowhead marks a tiny space of cytosol, whereas the asterisks indicate the apoplastic space of different cell cavities. Arrows within the bright field image indicate cell plasma membranes.

Rust-induced secreted protein carries a predicted N-terminal signal peptide for secretion (**Figure 1A**). In order to verify the functionality of the predicted signal peptide, a RISP-mCherry fusion was transiently expressed in *N. benthamiana* leaf pavement cells by the agro-infiltration method and the mCherry fluorescence was observed by live-cell imaging. A specific signal was detected around the cells. Plasmolysis experiments confirmed that the mCherry signal was exclusively observed in the apoplast (**Figure 1B**). We concluded that RISP signal peptide is functional and efficiently targets the protein to the apoplast.

### RISP Can be Overexpressed in *Escherichia coli* Cytosol and Purified as a Monomer

The predicted mature form of RISP was expressed in *E. coli* with an N-terminal hexahistidine tag (His-RISP) or without (RISP). The proteins were purified to homogeneity by affinity chromatography (His-RISP) or by ammonium sulfate fractionation, gel filtration, and ion exchange chromatography (RISP; Supplementary Figure S1A). Given the presence of four cysteine residues and the prediction that residues 48–70 and 55–79 would form disulfide bonds, we assessed the RISP redox state by thiol titration experiments. No thiol group was titrated in the purified protein, even under denaturing conditions, whereas 3.57 ± 0.35 free thiol groups per protein were titrated using a pre-reduced protein (Supplementary Figure S1B). This indicated that RISP was completely oxidized at the end of the purification. Mass spectrometry analyses of an untreated RISP protein identified a single peak corresponding to the size of a RISP monomer. The absence of disulfide-bridged oligomers indicated that the protein contained two intramolecular disulfide bridges.

**FIGURE 2:**
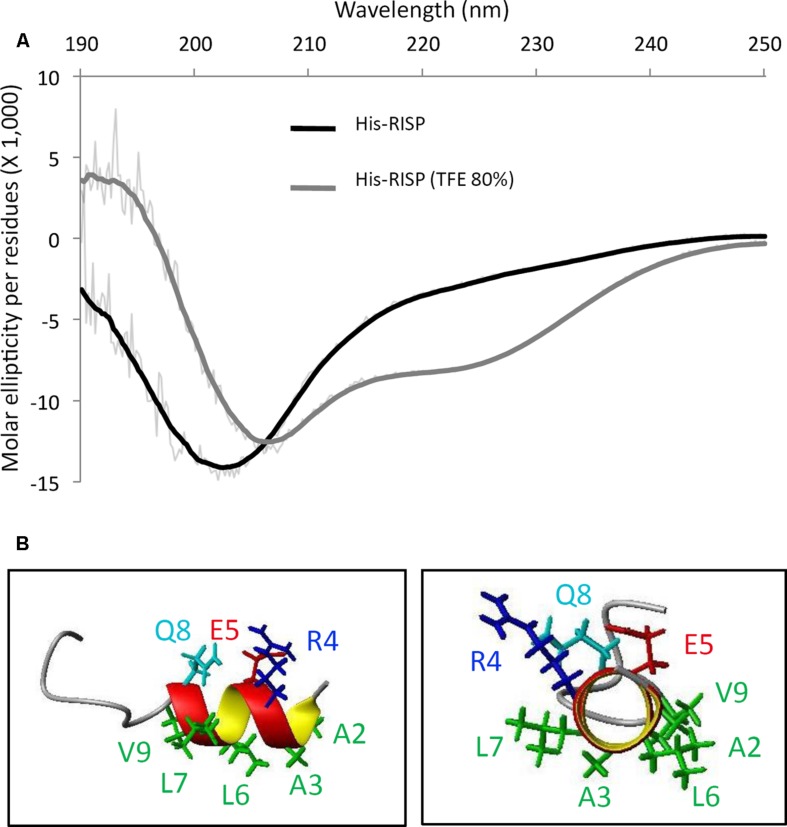
**RISP is intrinsically disordered and adopts α-helices in TFE. (A)** Circular dichroism (CD) spectra of His-RISP in aqueous solution (phosphate buffer pH 7.0, in black) or in 80% trifluoroethanol (TFE; in gray). Thick lines: smoothed curves (*n* = 25), thin lines: curves established with raw CD data. **(B)** Nuclear magnetic resonance (NMR) model of the 15 N-terminal residues of His-RISP in 80% TFE-*d*_3_/20% phosphate buffer pH 7.4, at 25°C. Side chains of residues 2–9 are represented in green (hydrophobic side chains), dark blue (positively charged side chain), red (negatively charged side chain) or light blue (polar side chain).

Rust-induced secreted protein structural properties have been further analyzed using circular dichroism (CD) and NMR spectroscopy. Although recombinant tagged and untagged RISP can be purified without any sign of precipitation or insolubility, the CD spectrum of a His-RISP indicated that the protein has no detectable secondary structure (**Figure 2A**). This was corroborated with the poor spectral dispersion and the very few NOESY correlations observed in the NMR spectra of a ^15^N-labeled RISP recorded in phosphate buffer at various pH values (4.9–7.0) and temperatures (15–50°C; Supplementary Figure S2A). The reduction of His-RISP had a negligible effect on the CD spectrum, which indicated that the disordered state of RISP is independent of the cysteine redox state (Supplementary Figure S2B). Interestingly, in the presence of 80% trifluoroethanol (TFE), a solvent classically used to promote the acquisition of protein secondary structures, the His-RISP CD spectrum was consistent with a partial folding; the protein showing up to 34% of predicted α-helices (**Figure 2A**; Supplementary Table S2). In 80% TFE, albeit most residues gave only broad unresolved peaks in the H^α^/amide region, some residues displayed numerous and relatively sharp NOESY correlations in the aliphatic/amide region as well as in the amide/amide region (Supplementary Figure S3). This has enabled the assignment of the first 15 residues. Residues 2–8 (AARELLQ, which correspond to residues 24–30 in **Figure 1A** including the leader sequence) appeared to show typical correlations for helical structure, i.e., medium-range correlations in the aliphatic/amide region, together with medium H^N^(i)- H^N^(i+1) and weak H^N^ (i)- H^N^ (i+2) correlation peaks in the amide-amide region (Supplementary Figure S3). The solution structure of the 15 assignable N-terminal residues of His-RISP was modeled using NMR distance restraints. Polar or charged side-chains of this helix appeared to be located on the opposite side to the hydrophobic ones (**Figure 2B**), resulting in an amphiphilic character. The presence of structural order limited to the N-terminal region was coherent with the prediction of secondary structure and of intrinsically disordered regions made by the Globplot software (**Figure 1A**, Supplementary Figure S3). The unfolded nature of the protein prompted us to investigate its thermosolubility. After 10 min incubation at 95°C, both His-RISP and RISP remained soluble. We concluded from this set of experiments that RISP is thermosoluble and in a disordered state in aqueous solution, but can acquire some secondary structures in specific conditions.

### RISP Binds to *M. larici-populina* Urediniospores and Inhibits Germination and Germ Tube Elongation *In Vitro*

**FIGURE 3:**
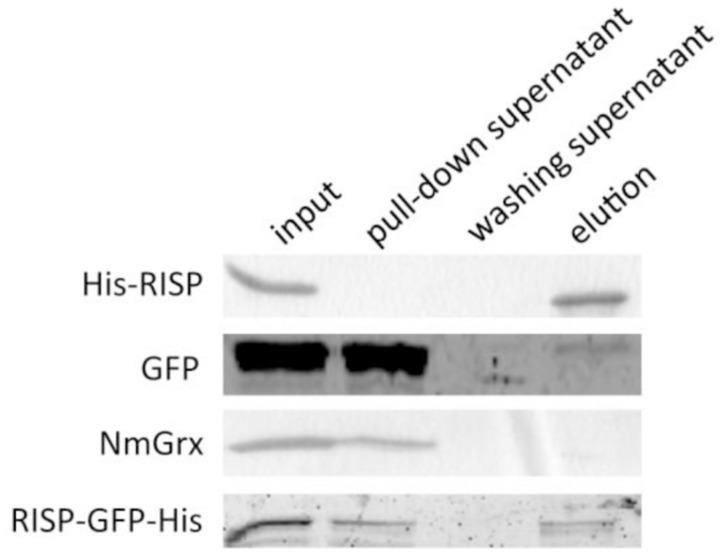
**RISP interacts with *M. larici-populina* urediniospores *in vitro.*** Recombinant His-RISP, RISP-GFP-His, GFP and *Neisseria meningitidis* Grx were included in a pull-down assay with *M. larici-populina* urediniospores (isolate 98AG31). Recombinant proteins from the different fractions of the pull-down assay were visualized on 15% SDS-PAGE. Input: protein solution before centrifugation. Pull-down supernatant: supernatant after the first centrifugation. Washing supernatant: supernatant after the second centrifugation for washing. Elution: supernatant after incubation at 95°C in Laemmli buffer.

Several eukaryotic AMPs are cationic and intrinsically disordered in aqueous solution. Their positively charged residues and amphipathic nature usually promote interaction with microbial structures (Melo et al., 2009). The fact that RISP has comparable properties prompted us to investigate whether RISP can attach to *M. larici-populina* urediniospores *in vitro*. We developed a pull-down assay by mixing urediniospores with RISP and with several recombinant proteins used as controls. His-RISP co-precipitated with urediniospores whereas other recombinant proteins, such as a GFP or a *Neisseria meningitidis* glutaredoxin, remained mostly in the supernatant (**Figure 3**). For visualizing the RISP-urediniospore interaction, we produced and purified a His-tagged GFP fusion protein (RISP-GFP-His). However, only very few RISP-GFP-His did co-precipitate with urediniospores (**Figure 3**). We concluded that RISP stably interacts with *M. larici-populina* urediniospores whereas the GFP-fusion only weakly binds spores, the GFP tag likely preventing full binding.

**FIGURE 4:**
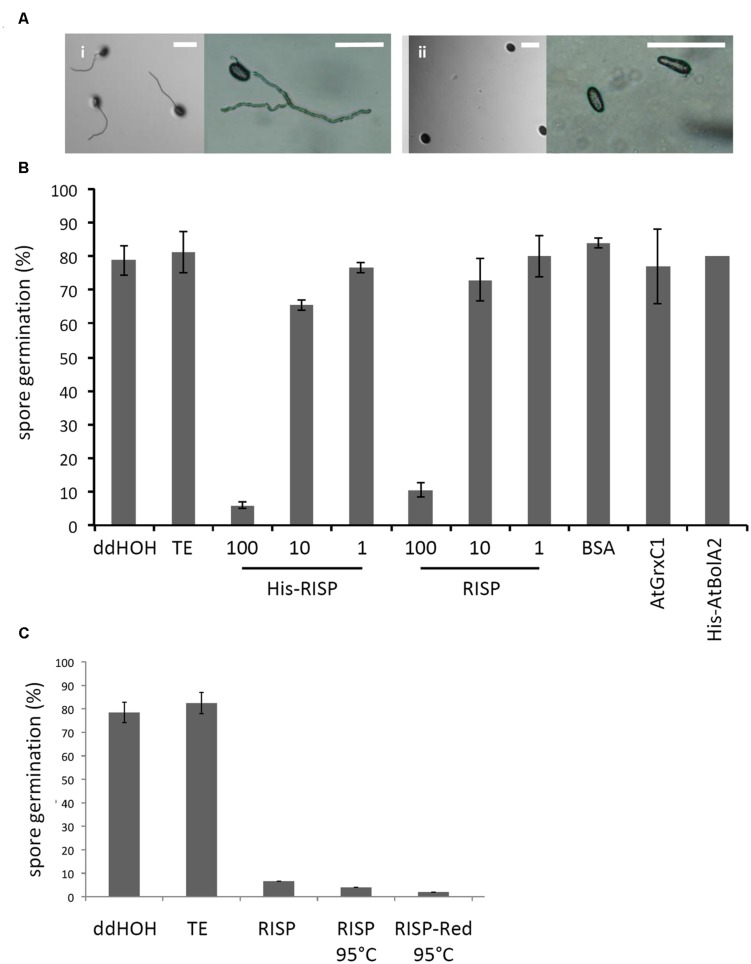
**RISP inhibits *M. larici-populina* spore germination *in vitro.* (A)** *M. larici-populina* urediniospores were deposited on water-agar Petri dishes with (i) TE pH 8.0 buffer (i.e., control without protein) or (ii) His-RISP and pictures were taken after 5 h. Scale bar: 100 μM. **(B)** *M. larici-populina* urediniospores were deposited on water-agar Petri dishes with RISP, His-RISP, BSA, AtGrxC1, His-AtBolA2, TE pH 8.0 buffer (i.e., control without protein) or ddHOH. After 5 h, a minimum of 300 urediniospores were observed and considered to calculate a percentage of germination. RISP concentration is indicated in μM. BSA, AtGrxC1, and His-AtBolA2 were used at a concentration of 100 μM. Error bar: SE, *n* ≥ 3 (His-AtBolA2, *n* = 2). **(C)** As in B, with 100 μM RISP, 100 μM RISP boiled for 10 min (RISP 95°C) or 100 μM RISP DTT-reduced and boiled for 10 min (RISP-red 95°C). Error bar: SE of independent repeats, *n* ≥ 2.

**FIGURE 5:**
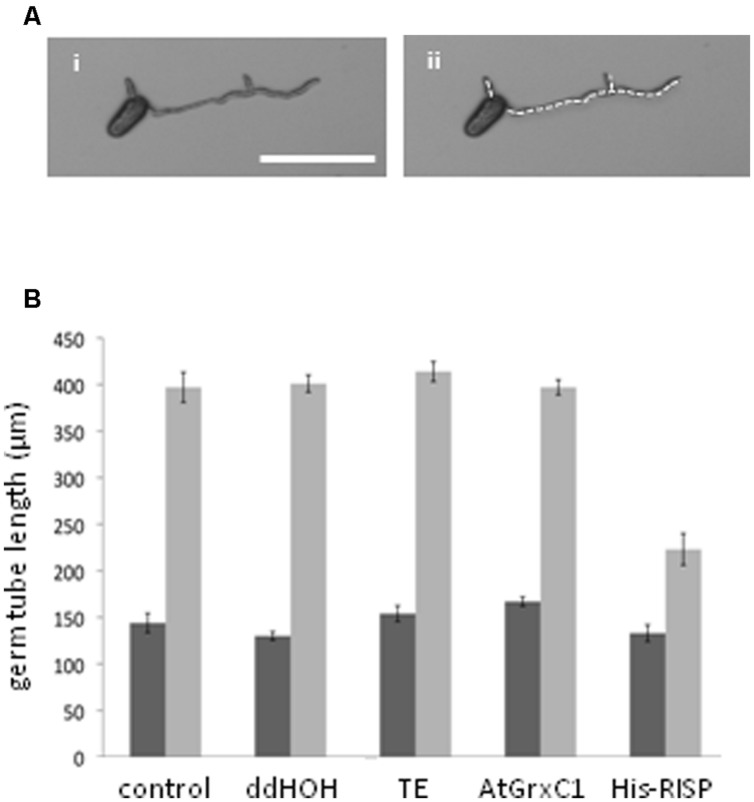
**RISP inhibits *M. larici-populina* germinating tube elongation *in vitro.* (A)** Pictures of *M. larici-populina* urediniospores that germinated 4 h on water-agar in Petri dishes. (ii) Same picture as (i) with the white dotted line marking the measured length of the germ tubes. Scale bar: 100 μM. **(B)** Inhibitory effect of RISP on the elongation of germinating tube *in vitro*. *M. larici-populina* urediniospores germinated for 4 h on water-agar in Petri dishes, then 100 μM His-RISP, 100 μM AtGrxC1, TE pH 8.0 buffer or ddHOH were carefully deposited onto the medium and left 4 additional hours. ‘Control’ indicates non-treated urediniospores. Pictures were taken under a binocular microscope 4 h (dark gray bars) and 8 h (light gray bars) after the beginning of the experiment and were used to measure the total length of germ tube(s) of a minimum of 15 spores for each repetition. Error bar: SE, *n* = 5.

Then, an antifungal assay was set up to evaluate the capacity of purified recombinant proteins to inhibit the germination of *M. larici-populina* urediniospores *in vitro* (**Figure 4A**). Both His-RISP and RISP impaired urediniospore germination at 10 μM and almost completely abolished germination at 100 μM (**Figures 4A,B**). In contrast, BSA or other recombinant proteins did not affect urediniospore germination (**Figure 4B**). The ability to inhibit urediniospore germination was still observed after heating oxidized or reduced recombinant RISP for 10 min at 95°C, indicating that its antifungal activity is insensitive to the temperature and independent of the protein redox state (**Figure 4C**). To assess whether RISP can also affect germ tube elongation, His-RISP was added to germinating urediniospores (incubated for 4 h) and germ tube length was measured after 4 additional hours (**Figure 5A**). His-RISP reduced germ tube elongation to 75% compared to controls (**Figure 5B**). We concluded that RISP possesses an antifungal activity *in vitro* against *M. larici-populina*.

We investigated whether RISP antifungal activity could be extended to other microbes by testing the ability of purified RISP to inhibit *in vitro* mycelium growth of the mycorrhizal basidiomycete *L. bicolor* and both mycelium growth and spore germination of the rice blast ascomycete *M. oryzae*. However, RISP neither inhibited the growth nor the germination of these fungi (Supplementary Figure S4). This suggests that RISP antifungal activity is not effective against a wide range of fungi and may be specifically targeted to *M. larici-populina*.

### RISP Inhibits *M. larici-populina* Growth on Poplar Leaves

**FIGURE 6:**
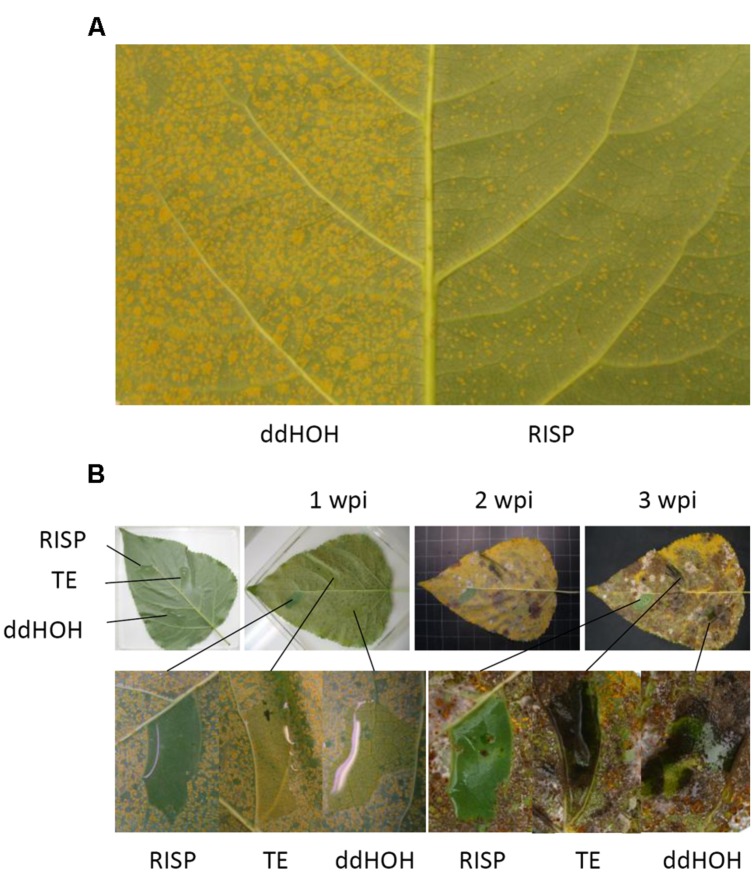
**RISP inhibits *M. larici-populina* growth on poplar leaves. (A)** The left part of a Beaupré leaf was inoculated with *M. larici-populina* urediniospores (isolate 98AG31, virulent on Beaupré) mixed with TE pH 8.0 buffer, whereas the right part was inoculated with the same batch of urediniospores mixed with 100 μM recombinant RISP. Pictures were taken at 10 dpi. **(B)** Selected areas of a Beaupré leaf were treated with 1 mL of ddHOH, TE pH 8.0 buffer or 100 μM RISP, and then the whole leaf was immediately inoculated by pulverization with *M. larici-populina* urediniospores (isolate 98AG31). Pictures were taken just after inoculation as well as 1, 2, or 3 weeks post-inoculation (wpi).

To further validate the antifungal activity observed *in vitro* and to assess whether RISP can inhibit *M. larici-populina* growth directly on leaves, purified RISP was applied simultaneously with urediniospores of a virulent isolate onto poplar leaves. The level of fungal growth was then evaluated by analyzing the final number of uredinia, i.e., orange pustules containing newly formed urediniospores, produced after 10 days on the leaf surface. As shown in **Figure 6A**, RISP strongly inhibited fungal development compared to the control. As an alternative method, RISP was locally deposited on specific leaf areas and the whole leaf was inoculated with urediniospores either immediately or after 3 or 5 days. In all cases, RISP-treated areas remained free of uredinia up to 3 weeks after inoculation (**Figure 6B**; Supplementary Figures S5A,B). Similar results have been obtained with His-RISP (Supplementary Figure S5C). We concluded that RISP presents an antifungal activity against *M. larici-populina* both *in vitro* and on the surface of poplar leaves.

### RISP Induces Poplar Cell Culture Alkalinisation

**FIGURE 7:**
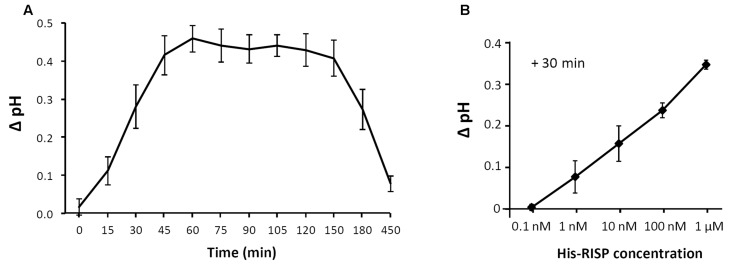
**RISP induces poplar cell culture alkalinisation. (A)** His-RISP was added to poplar cell suspensions (*P. trichocarpa* x *P. deltoides* H11-11 hybrid) at a concentration of 1 μM and the pH was recorded at regular times as indicated. A control without protein was performed with TE pH 8.0 buffer. Error bars: SE, *n* = 3. **(B)** His-RISP was added to the H11-11 cell suspensions at concentrations ranging from 0.1 nM to 1 μM and the pH was recorded after 30 min. Error bars: SE, *n* = 3. TE buffer values (stable at pH 4.2 during the experiment) were used to calculate the pH ratio for each time-point.

The recent observation that the *RISP* gene is adjacent to a *LRR-RLP* gene in the poplar genome sequence and that both genes are co-expressed upon *M. larici-populina* infection led to the hypothesis that RISP may function as an elicitor of cellular responses through this specific receptor-like protein (Petre et al., 2014). To test the ability of RISP to elicit a plant response, we used a poplar cell culture medium alkalinisation assay (Haruta and Constabel, 2003). When added to poplar cell cultures at a final concentration of 1 μM, His-RISP induced a shift of 0.5 pH units after 45 min whereas no pH shift was observed with the buffer alone (**Figure 7A**). An alkalinisation was still observed by decreasing His-RISP concentration to 1 nM, although the pH shift was lower (**Figure 7B**). To rule out the possibility that an *E. coli* contaminant co-purified with the protein triggered cell culture alkalinisation, the assay was repeated with a concentrated protein extract consisting of IMAC-retained proteins from the *E. coli* production strain (Supplementary Figure S6). These contaminants did not affect the pH of the cell culture, indicating that RISP specifically induced cell culture alkalinisation. We concluded that RISP is able to rapidly elicit plant cell responses in a dose-dependent manner.

### RISP Transcript Accumulation During Poplar Immune Responses to *M. larici-populina* is Not Reflected at the Protein Level

**FIGURE 8:**
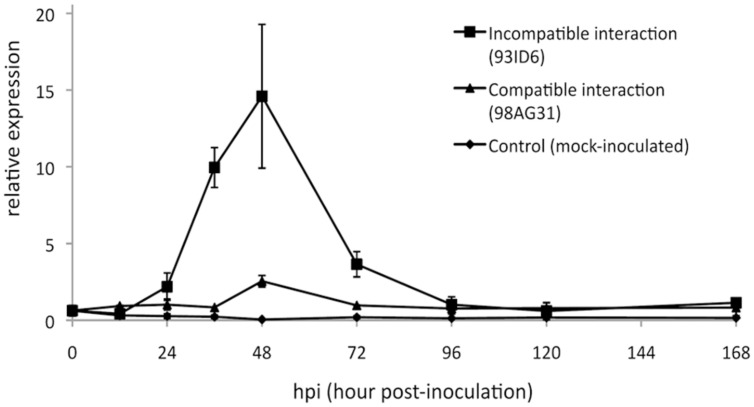
**RISP transcripts accumulate in poplar leaves during immune responses.** Reverse transcription-quantitative polymerase chain reaction (RT-qPCR) expression profile of *RISP* transcripts during a time-course infection of ‘Beaupré’ leaves with either virulent (compatible interaction) or avirulent (incompatible interaction) isolates of *M. larici-populina*. Mock-inoculated leaves were used as a control treatment and transcript expression was normalized to a poplar ubiquitin transcript. Error bar: standard deviation of the values from two biological replicates.

Previous analyses showed that *RISP* transcripts strongly accumulate at 48 h post-inoculation (hpi), a time-point corresponding to the establishment of poplar defense responses and to the arrest of the growth of an avirulent isolate of *M. larici-populina* (Rinaldi et al., 2007). We used RT-qPCR to further examine *RISP* transcripts accumulation during a complete infection cycle on poplar leaves with virulent and avirulent isolates of *M. larici-populina*. This time-course included key time-points such as 12 hpi (penetration through the stomata), 24 hpi (formation of the first haustorial infection structures), 48 hpi (arrest of avirulent isolate growth), and 168 hpi (formation of uredinia symptoms by the virulent isolate and urediniospores release) (Hacquard et al., 2011). In leaves infected with the avirulent isolate, *RISP* transcripts strongly accumulated between 36 and 72 hpi, with a peak of expression at 48 hpi (**Figure 8**). In contrast, transcript levels remained basal along the whole time-course experiment in either mock-inoculated leaves or leaves infected by a virulent isolate. Hence, by refining the expression pattern of *RISP*, we confirmed that it is specifically induced in poplar leaves during immune responses against *M. larici-populina*, as previously reported (Rinaldi et al., 2007; Petre et al., 2014).

**FIGURE 9:**
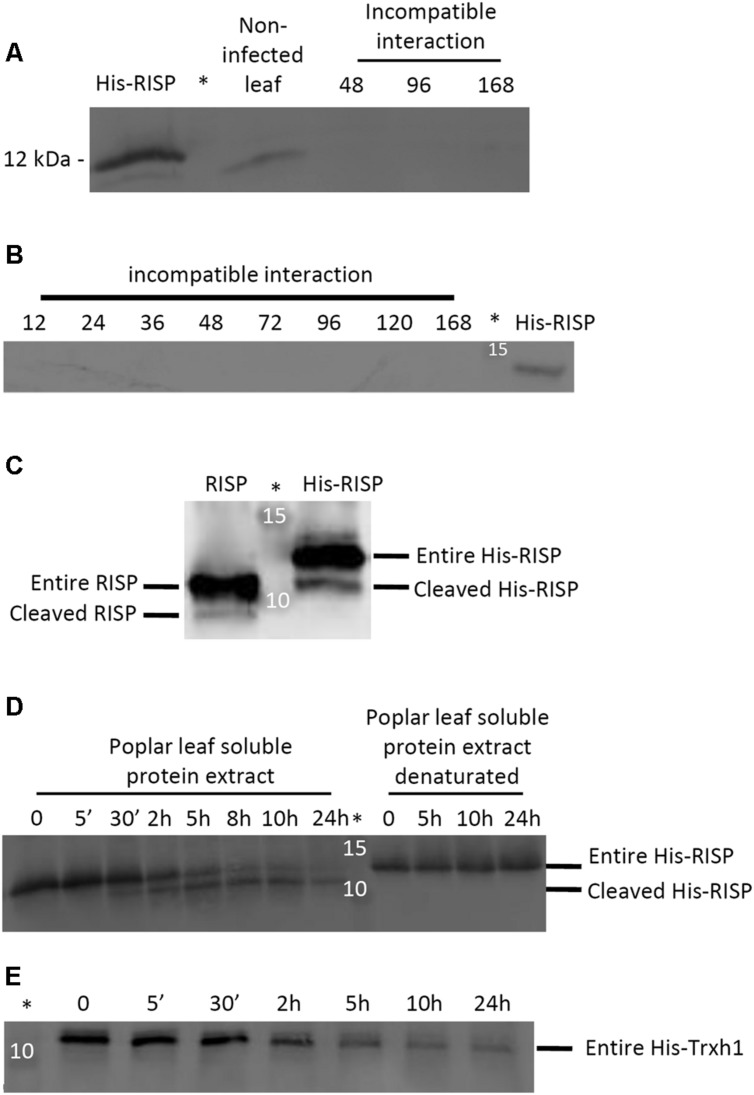
**RISP is not detected in poplar leaves during immune responses and its C-terminus is cleaved by a plant-encoded mechanism. (A)** Western blot showing the expression of RISP in non-infected poplar leaves and at three time-points of poplar leaves during an incompatible interaction with *M. larici-populina* (see main text for details). **(B)** Western blot showing the expression of RISP over the whole time-course experiments of poplar leaves during an incompatible interaction with *M. larici-populina*. In A and B, proteins isolated from poplar leaves (30 μg: 15 μg of soluble proteins + 15 μg of insoluble proteins) were separated on 15% SDS-PAGE and transferred on nitrocellulose membrane. The His-RISP recombinant protein (20 ng in A and 10 ng in B) was used as a reference. Anti-RISP polyclonal antibodies were used for primary detection of RISP. Numbers presented indicate hpi. **(C)** Five percent (w/w) of purified RISP and His-RISP were incubated with soluble protein extracts from poplar leaves for 30 min at room temperature under gentle agitation and with 1 mM phenylmethanesulfonylfluoride (PMSF). **(D)** RISP time-course incubation with soluble protein extracts from poplar leaves. Five percent (w/w) of purified His-RISP was incubated with untreated or denatured soluble protein extracts from poplar leaves for up to 24 h at room temperature under gentle agitation and with 1 mM PMSF. **(E)** His-tagged thioredoxin h1 (His-Trxh1) time-course incubation with soluble protein extracts from poplar leaves. Five percent (w/w) of purified His-Trxh1 was incubated with soluble protein extracts from poplar leaves for up to 24 h at room temperature under gentle agitation and with 1 mM PMSF. Protein integrity was followed by western blot. ^∗^Indicates the lane occupied by the molecular weight marker.

In order to test whether RISP also accumulates at the protein level during an incompatible interaction with *M. larici-populina*, we performed western blotting experiments with a polyclonal antibody raised against the recombinant His-RISP. The antibody efficiently labeled the recombinant His-RISP as well as the endogenous RISP in a total leaf protein extract consisting of an equal mixture of soluble and insoluble proteins (**Figure 9A**). However, RISP was surprisingly not detected in poplar leaves over a time-course infection with an avirulent isolate (**Figures 9A,B**). All subsequent attempts that aimed at identifying RISP by western blot during the incompatible interaction, in particular between 48 and 96 hpi, failed (e.g., by extracting total proteins in a denaturing buffer, by adding a protease inhibitor cocktail, or by performing an enrichment with soluble and thermostable proteins; Supplementary Figure S7). The anti-RISP antibody was also used in co-immunoprecipitation experiments from soluble proteins isolated from *M. larici-populina*-infected poplar leaves as well as immunolocalisation experiments performed on the same tissues (data not shown). In all cases, we were unable to detect RISP at the protein level. This indicated that RISP transcript and protein levels do not correlate during immune responses to *M. larici-populina*, raising the question of a possible modification (degradation or cleavage) of RISP in leaves.

### RISP C-Terminus is Cleaved by a Plant-Encoded Mechanism

An approach that has been successfully used to detect the cleavage of protein precursors is to incubate them with plant protein extracts (Guo et al., 2011). To determine whether RISP can be subject to a plant-driven processing, purified RISP and His-RISP were incubated with soluble proteins isolated from poplar leaves, and their integrity assessed by western blot. After 30 min of incubation, a band with a smaller apparent molecular mass appeared for both RISP and His-RISP (**Figure 9C**). In order to better delineate the dynamics of RISP cleavage, the time-course incubation was extended over 24 h with His-RISP. Following 30 min of incubation, the lower band gradually appeared concomitantly with the disappearance of the upper band corresponding to the entire protein (**Figure 9D**). Incubation with heat-denatured soluble protein extracts prevented His-RISP cleavage, suggesting that it was triggered by some thermo-sensitive plant protease activity. Since both untagged and N-terminally His-tagged proteins were cleaved, we favored the idea that the cleavage occurred at the C-terminus. To ensure that the lower band observed with His-RISP was not due to the cleavage of the His-tag, we used an N-terminally His-tagged thioredoxin h1 (His-Trxh1) as a negative control (**Figure 9E**). As a complementary experiment, we have re-purified a mixture of the entire and processed forms of His-RISP using IMAC. After SDS-PAGE separation, the band corresponding to the processed form was digested by trypsin and analyzed by mass spectrometry. Peptides encompassing the His-tag have been unambiguously identified in this sample (data not shown). From these experiments, we concluded that a thermo-sensitive agent (likely a protease) present in poplar leaves promoted the cleavage of the C-terminal end of RISP.

## Discussion

In this study, we reported the biochemical, structural, and functional characterisation of the RISP from poplar. We showed that RISP is secreted into the apoplast when transiently expressed in *N. benthamiana* leaf pavement cells. The predicted N-terminal signal peptide of RISP probably mediates the targeting to the extracellular space through the ER-Golgi secretory pathway. The absence of a fluorescent signal in the cytosol and of any sign of aggregation in the ER-Golgi suggests an efficient secretion. The apoplastic localisation is particularly suited for the two demonstrated roles of RISP, i.e., inhibition of the growth of the fungal pathogen *M. larici-populina*, and elicitation of cellular responses. The dual function of RISP is further discussed below.

### RISP Possesses an Antifungal Activity Against *M. larici-populina*

We have shown that RISP co-precipitates with *M. larici-populina* urediniospores in pull-down assays and inhibits their germination. It is noteworthy that the inhibitory effect of RISP on spore germination was used as a proxy to detect its antifungal activity, but may not happen upon natural infections. Indeed, *RISP* expression is induced at 48 hpi by an avirulent strain, a time-point at which fungal structures have already developed inside the leaves. The only possibility is that enough RISP protein persists on leaves until another cycle of infection. For this reason, an assay measuring the capacity of RISP to inhibit the elongation of germinating tubes (a structure sharing the topology of infection hyphae exploring the leaf apoplast during infections) has been set up. It revealed that RISP was also able to inhibit the elongation of *M. larici-populina* germinating tubes *in vitro*. Altogether, these findings suggest that, during leaf infection, RISP is likely secreted in the apoplast where it can directly interact with fungal structures to restrict their development.

An important issue in these bioassays is the concentration of RISP (100 μM) that fully prevented *M. larici-populina* urediniospore germination, since it is in the upper range of what can be found in the literature about plant antifungal proteins (Silva et al., 2014). It is worthy to mention that the indicated concentrations correspond to the initial concentrations of the protein stock solution. For instance, the use of solid media through which proteins diffuse prevents us from rigorously determining the exact concentration of RISP active in these assays. For this reason, the RISP concentration exerting an antimicrobial activity is likely much lower. Concerning the co-inoculation experiments of the virulent strain of *M. larici-populina* with RISP on poplar leaves, it is first important to specify that the absence of rust disease symptoms is likely attributed to the fact that RISP inhibits the germination of urediniospores present on the surface of the leaves as observed in the *in vitro* system. Other important aspects to consider are that high spore concentrations are used and that a single spore is sufficient to generate an infection and produce a uredinia. Thus, the fact that we observed no lesions on leaves treated with 100 μM RISP suggests that RISP prevented the germination of almost 100% of the spores, which was never observed in the tests performed *in vitro*. Hence, RISP seems to be a more potent inhibitor of spore germination on poplar leaves. A possible explanation is that part of the protein can diffuse through the wax and prevent the growth of the fungal structures that have invaded the leaf, either directly as observed *in vitro* through the inhibition of germ tube elongation or by eliciting some cellular responses. Nevertheless, this explanation is unlikely considering the existence of a very thick and impermeable cuticle in leaves of this poplar cultivar. A more plausible explanation is that contrary to *in vitro* experiments where protein solutions are rapidly diluted into the agar, the RISP solution remains as a drop on leaves and spores are continuously exposed to a high RISP concentration for hours.

Known plant AMPs are usually structured proteins (Pelegrini et al., 2011) whereas we have observed that RISP is in a disordered state in aqueous solutions (**Figure 2A**). The fact that it can partially fold in the presence of TFE suggests that RISP might fold in particular conditions, for example in contact with target molecules. From the co-precipitation assay with *M. larici-populina* urediniospores (**Figure 3**) some external molecules present at the surface of the urediniospores constitute one of these targets. The disruption of membrane integrity by AMPs can be due to the formation of toroidal pores formed by a supramolecular assembly of peptides and lipids, as well as to the accumulation of high concentrations of peptides whose amphipathic structures align parallel to the membrane surface (Bechinger and Salnikov, 2012). In accordance with this need to form structures with a particular arrangement, we observed that the presence of a GFP tag precluded RISP interaction with the spores.

### RISP Elicits Cellular Responses Upon Incubation with Poplar Cells

Besides its antifungal properties, the genomic proximity and co-regulation of the *RISP* gene with a gene encoding a LRR-RLP prompted us to investigate a possible elicitor function (Petre et al., 2014). Cell-culture alkalinisation assays have been widely used to identify and characterize plant elicitors in a number of species (Pearce and Ryan, 2003; Scheer et al., 2003; Huffaker et al., 2006; Pearce et al., 2009, 2010; Chang et al., 2013). Haruta and Constabel (2003) previously reported the use of the poplar cell line H11-11 (*P. trichocarpa* x *P. deltoides*) to characterize poplar rapid-alkalinisation factor 1 (RALF1). The alkalinisation profile observed with His-RISP is similar to the one previously reported for RALF1, reaching a maximal alkalinisation of approximately 4.7 pH units. Maximal cell alkalinisation was obtained with a concentration of 1 μM. This concentration is higher than those usually reported for RALF or other peptides in cell cultures from different plant species (Haruta and Constabel, 2003; Yamaguchi et al., 2011) but we noticed that an effect is still visible at 1 nM with His-RISP (**Figure 7B**).

One hypothesis that may explain the requirement for higher concentrations is that the RISP form used in these experiments is not similar to the native protein, i.e., the one produced *in planta*. For instance, while disulfide bond formation into inceptin has no effect on its biological activity (Schmelz et al., 2007), it was observed that the *in vitro* oxidation of a synthetic tobacco RALF peptide leads to the formation of different oxidized forms that are more or less active (Pearce et al., 2001). Considering the presence of four cysteines in RISP, variations in its redox state may explain this difference. However, RISP structure and antifungal activity is not affected by disulfide bond reduction. Another hypothesis to explain the high concentration of RISP required for these elicitor activity assays is that we have used a precursor protein and not the bioactive peptide. It is well-documented that peptide hormones are often released from their precursors upon cleavage (Germain et al., 2006; Matsubayashi, 2014). Consistently, we have observed that a plant mechanism promoted the specific cleavage of several amino acids from the C-terminus of RISP. In parallel, the absence of RISP detection in infected poplar leaves, whereas the polyclonal anti-RISP antibodies efficiently detected nanograms of recombinant proteins, also supports the view that RISP is a cleavable precursor. Of course, we cannot completely exclude that other factors prevented RISP detection. Secreted peptide hormones might undergo post-translational modifications (Matsubayashi, 2014). In the case of RISP, it may mask the recognized epitopes. It may also be that protein accumulation occurs at spatially restricted sites making it almost undetectable using destructive approaches (Caillaud et al., 2013). However, we were unable to detect any specific signal in intact infected tissues using fluorescent immunolocalisation.

Although the cell culture alkalinisation assay revealed that RISP is able to trigger cellular responses, the nature of these responses remains unknown. In model plants such as *Arabidopsis thaliana*, alkalinisation assays are often associated with other bioassays such as MAP-kinase activation, ROS formation, callose deposition or activation of defense genes (Schwessinger et al., 2011; Lloyd et al., 2014). However, such assays cannot be established in our poplar species primarily because gene activation measurement in response to RISP is prevented by the difficulty to perform appropriate infiltration of poplar leaves. Note also that no or very low amounts of ROS are produced during poplar defense responses (Rinaldi et al., 2007). Hence, future investigations could rather consist in evaluating RISP elicitor activity in model plant species ectopically expressing the candidate LRR-RLP receptor associated with RISP (Petre et al., 2014).

## Conclusion

Rust-induced secreted protein combines both antimicrobial and elicitor activities. Growing evidence suggest that many AMPs of animals, fungi and plants are multifunctional peptides, which fulfill other biological functions related to immunity or not (Hegedus and Marx, 2013; Woriedh et al., 2015). For instance in animals, defensins are well-known AMPs with a wide range of secondary functions (Selsted and Ouellette, 2005; Yeung et al., 2011). In plants, *Capsicum annuum* antimicrobial protein 1 or *Prunus domestica* PR5-1 stimulate defense responses when ectopically over-expressed in *A. thaliana* (El-kereamy et al., 2011; Lee et al., 2011). Also, Chang et al. (2013) recently isolated a 16 amino acid peptide from sweet potato leaves that is able to induce tomato cell culture alkalinisation. This peptide corresponds to the N-terminus of a thaumatin-like protein (Chang et al., 2013), an antimicrobial protein belonging to the PR5 family (Petre et al., 2011). Future work should aim at determining the nature of the cellular responses triggered by RISP, the hypothesis being that these responses participate in immunity against *M. larici-populina*. Contrasting with most of the AMPs and peptide hormones that are often conserved in plants, there is no protein similar to RISP in other plant species. The study of RISP may reveal novel mechanisms that poplar developed to resist *M. larici-populina*.

## Author Contributions

BP, HG, AS, SD, and NR conceived and designed the experiments; BP, AH, HG, PT, JS, GP, SD, and NR performed the experiments and analyzed the data; BP, SD, and NR wrote the manuscript.

## Conflict of Interest Statement

The authors declare that the research was conducted in the absence of any commercial or financial relationships that could be construed as a potential conflict of interest.

## Footnotes

1 http://www.phytozome.net/poplar

2 href="http://multalin.toulouse.inra.fr/multalin/

3 http://www.genome.jp/tools/clustalw/

4 href="http://globplot.embl.de

5 http://www.cbs.dtu.dk/services/SignalP-3.0/

6 http://web.expasy.org/protparam/

7 http://clavius.bc.edu/~clotelab/DiANNA/

8 http://synbio.tsl.ac.uk/

9 href="http://fiji.sc/Fiji
