# Supplementary material for: The Poplar Rust-Induced Secreted Protein (RISP) Inhibits the Growth of the Leaf Rust Pathogen Melampsora larici-populina and Triggers Cell Culture Alkalinisation
Source: Front Plant Sci. 2016 Feb 17;7:97. doi: 10.3389/fpls.2016.00097 (PMC4756128; doi:10.3389/fpls.2016.00097)
Supplement: Supplementary file 1 [file Presentation_1.PDF]

SUPPLEMENTARY ONLINE DATA

The poplar Rust-Induced Secreted Protein (RISP) inhibits the growth of the leaf rust pathogen *Melampsora larici-populina* and triggers cell culture alkalinisation" by Petre et al

FIGURE LEGENDS

Figure S1. Production, purification and thiol titration of RISP recombinant proteins

(A) Ten micrograms of purified His-RISP or RISP were separated by 15% SDS-PAGE and detected by Coomassie Blue staining around 13 kDa (His-RISP) and 11 kDa (RISP) instead of their expected molecular weight (8.4 kDa and 6.2 kDa, respectively).

(B) Thiol titration of His-RISP either untreated or after DTT reduction.

Figure S2. RISP is intrinsically disordered in a redox-independent manner

(A) ^{1}H-^{15}N HSQC spectrum of ^{15}N-labelled His-RISP in 50 mM phosphate buffer (90% H_2O/ 10% D_2O) at pH 7.0 and 20°C.

(B) Circular dichroism (CD) spectra of oxidized (in black) and DTT-reduced (in grey) His-RISP. Thick lines: smoothed curves (n=25), thin lines: curves established with raw CD data.

Figure S3. RISP adopts α-helices in TFE

(A) Aliphatic/amide region of the 300 ms-NOESY spectrum of His-RISP in 80% TFE-d_3/20% phosphate buffer pH 7.4 at 25°C. Intra-residual correlations are represented by boxes and examples of $H^{\alpha}(i)/H^{N}(i+1)$ correlations by arrows.

(B) Amide/amide region of the 300 ms-NOESY spectrum of His-RISP in 80% TFE-d_3 / 20% phosphate buffer pH 7.4, at 25°C.

(C) His-RISP secondary structure in 80% TFE-d_3/20% phosphate buffer as determined by NMR. Only residues 1-15 could be assigned. The figure has been generated by CcpNmr.

Figure S4. Purified RISP does not affect *Laccaria bicolor* and *Magnaporthe oryzae* growth *in vitro*

(A) Mycelium growth inhibition assay. Drops of 10 µL of 100 µM RISP, TE pH 8.0 or ddHOH were deposited directly onto the medium or on sterile paper disk on the periphery of the mycelium of *L. bicolor* and pictures were taken 8 days later (a). Close up from a different plate are depicted in (b-c) as well as light microscope observation (40x) of the area in (d-e). A similar assay has been performed for *M. oryzae* (see Material and methods for details, data not shown) with similar results.

(B) Inoculum growth inhibition assay. Two mm^3 explants from *M. oryzae* (a) and *L. bicolor* (b) growing mycelia were transferred on fresh medium and completely covered with 100 µM RISP, TE pH 8.0 or ddHOH (or not treated as a control). Pictures were taken after 8 days.

(C) *M. oryzae* spore germination inhibition assay. Spores of *M. oryzae* were placed in small glass cupules in solutions with 100 µM His-RISP, TE pH 8.0 or ddHOH and pictures were taken after 24 hours.

Figure S5. RISP recombinant proteins inhibit *Melampsora larici-populina* growth on the surface of poplar leaves

(A-B) Selected areas of a Beaupré leaf were treated with 1 mL of ddHOH, TE pH 8.0 or 100 µM RISP, and then the whole leaf was inoculated after three days (A) or five days (B) by pulverisation with *M. larici-populina* urediniospores (isolate 98AG31). Pictures were taken just after inoculation as well as one to two weeks-post inoculation (wpi).

(C) Same experiment as described in Figure 6A, but with His-RISP instead of recombinant RISP, and with pipet inoculation on leaf disks.

Figure S6. IMAC-retained *Escherichia coli* proteins do not affect poplar cell culture pH

Twenty µL of TE pH 8.0 or concentrated purified IMAC-retained *E. coli* proteins (contaminants) were added to the H11-11 cell suspensions as described in Figure 7. The pH was recorded after 0, 30, and 60 min. Error bars: SE, n=4.

Figure S7. RISP is not detected in poplar leaves during immune responses to an avirulent isolate of *Melampsora larici-populina*

Twenty nanograms of recombinant His-RISP and fourty micrograms of total protein from poplar leaves 48h after inoculation with an avirulent isolate of *M. larici-populina* (I48) extracted as described in material and methods, extracted with a cocktail of protease inhibitors (Sigma Aldrich, Saint-Louis, USA) or with extracts from 1 g of leaf crude extract boiled prior to protein extraction in order to enrich in thermosoluble proteins were separated on 15% SDS-PAGE. Anti-RISP polyclonal antibodies were used for primary detection of RISP in the extracts by western blotting.

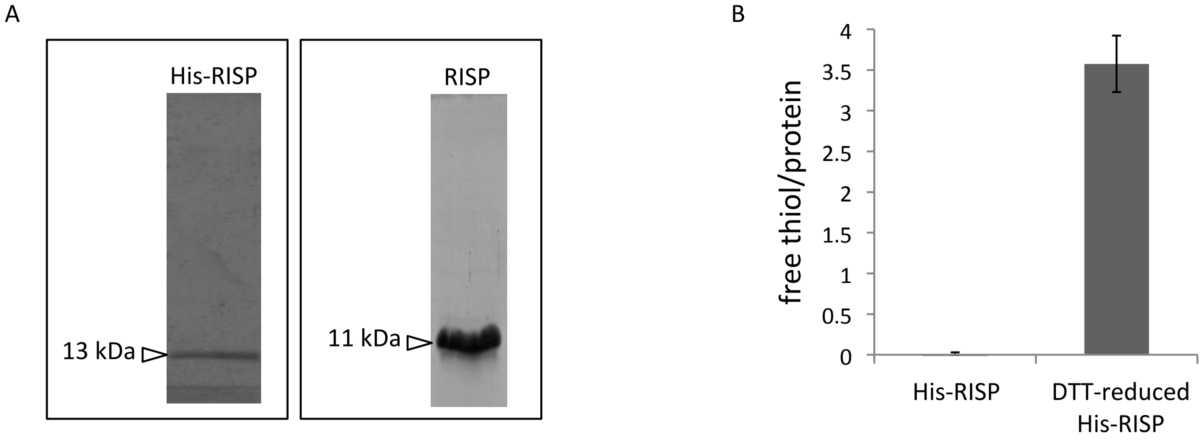

Supplementary Figure S1

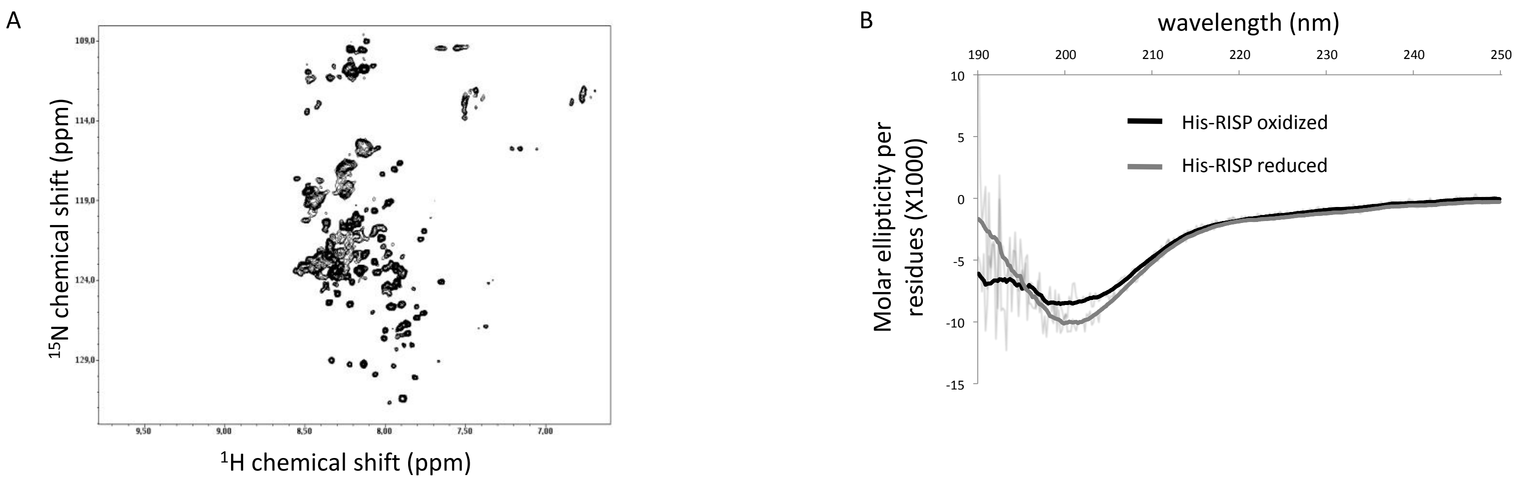

Supplementary Figure S2

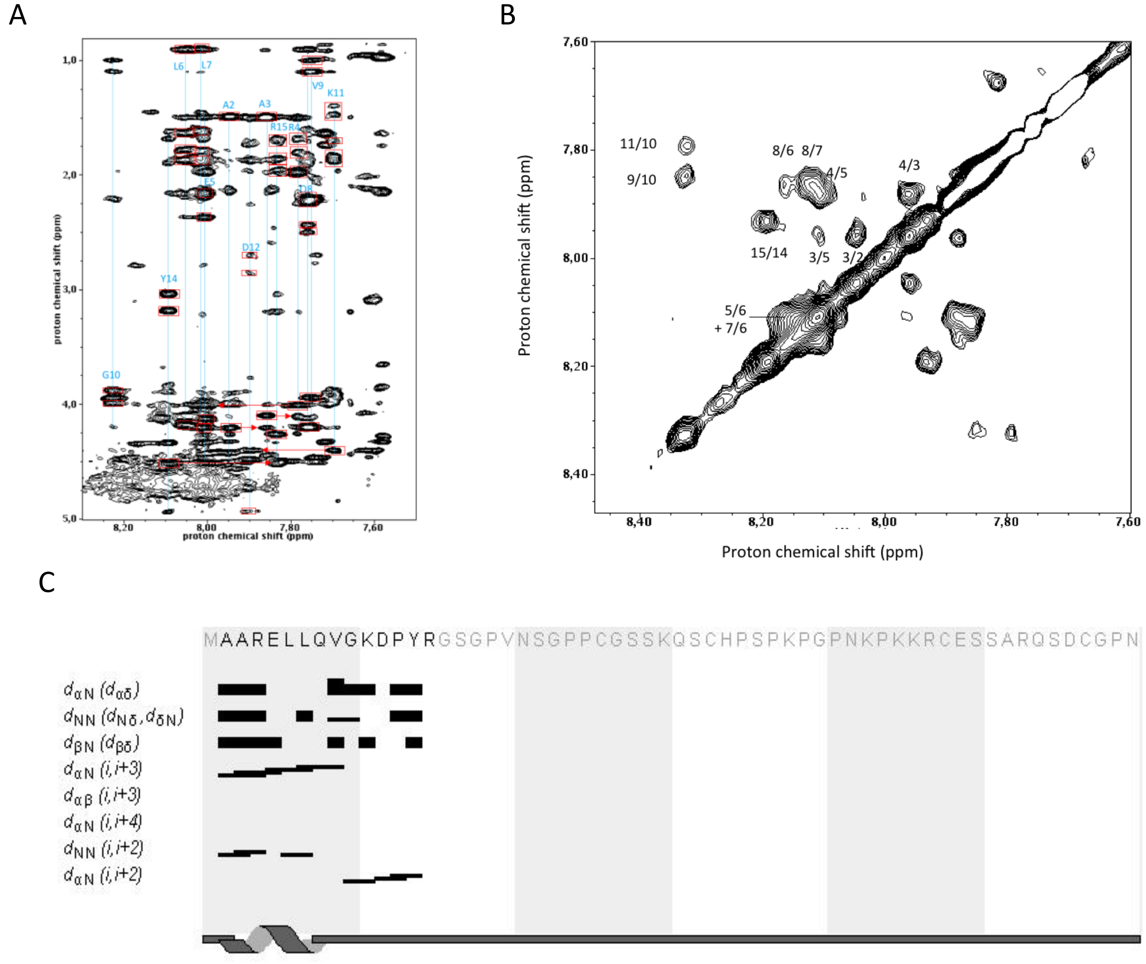

Supplementary Figure S3

A

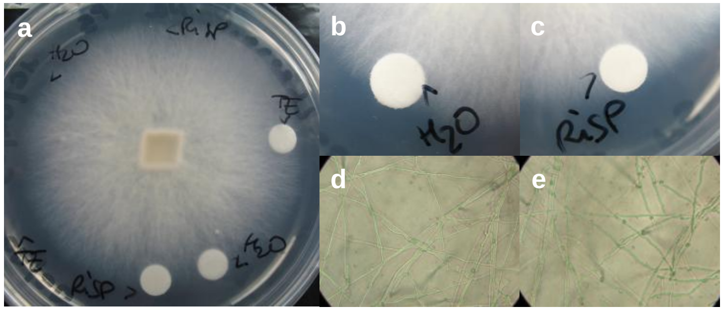

B

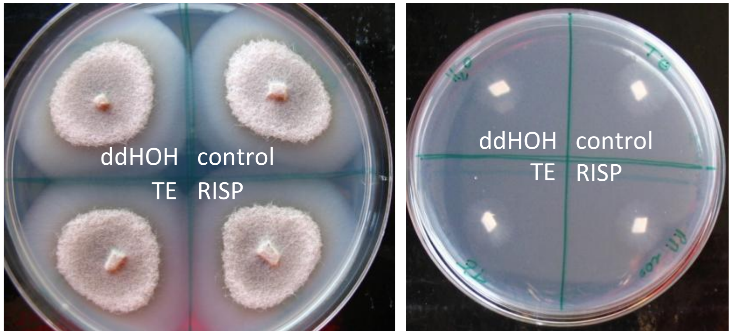

C

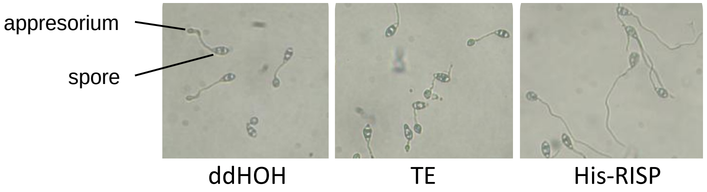

Supplementary Figure S4

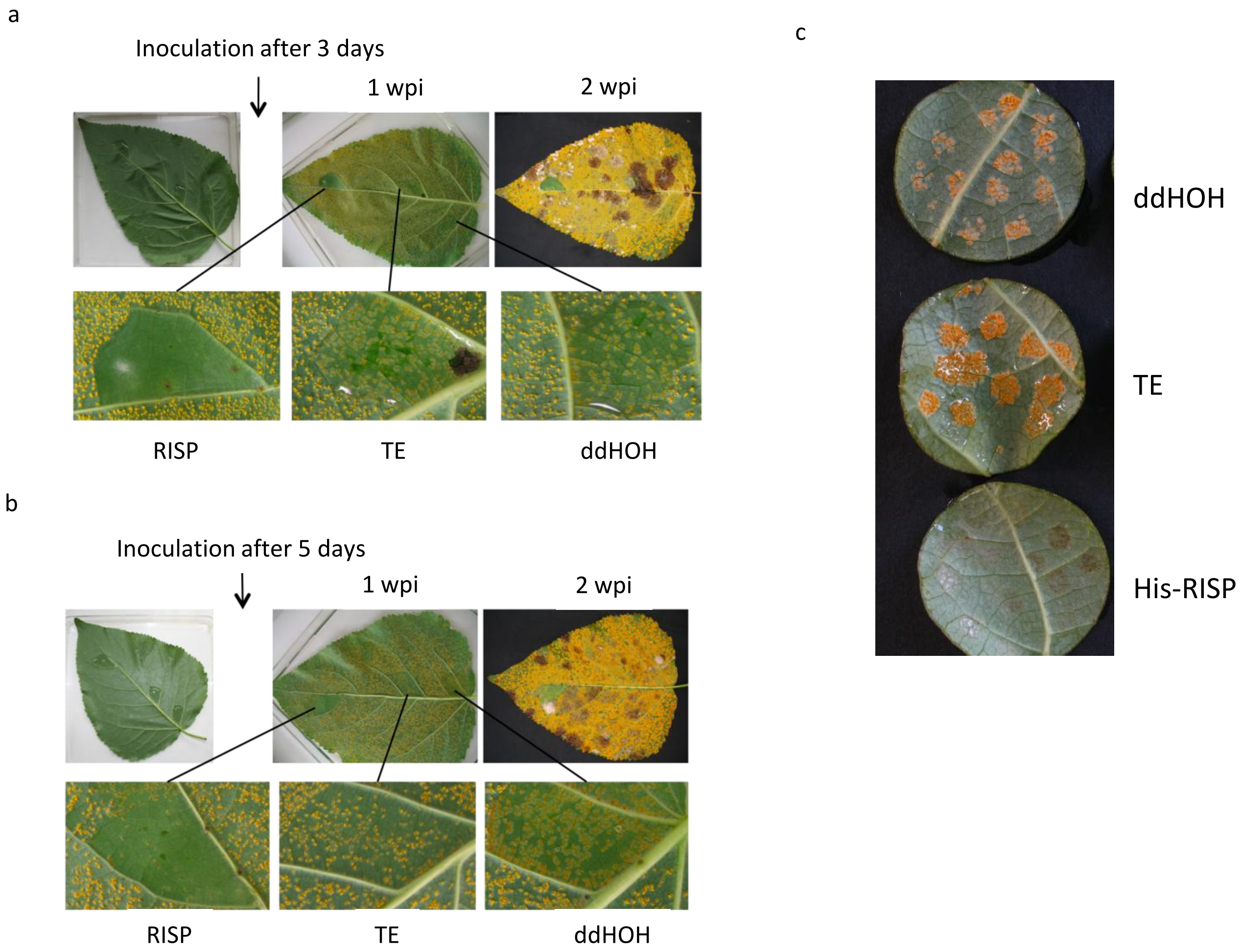

Supplementary Figure S5

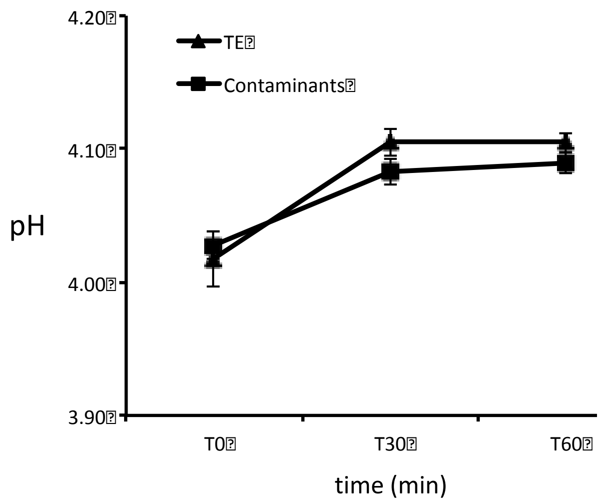

Supplementary Figure S6

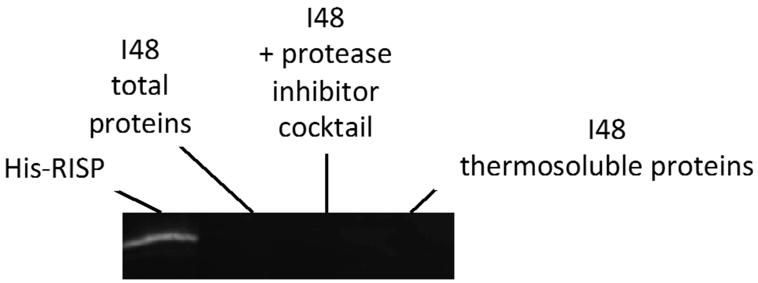

Supplementary Figure S7

Table S1. Primers, plasmids and protein sequences

Construct	Primer forward	Primer reverse	Plasmid	Expressed protein (His, GFP-His or mCherry tags are underlined)
RISP	5’-ccccCCATGGCTGCTCGAGAATTACTG-3’	5’-ccccGGATCCTCAATTTGGGCCACAATC-3’	pet3d	MAARELLQVGKDPYRGSGPVNSGPPCGSSKQSCHPSPKPGPNKPKKRCESSARQSDCGPN*
His-RISP	5'-ccccCCATATGGCTGCTCGAGAATTA-3'	5’-ccccGGATCCTCAATTTGGGCCACAATC-3’	pet15b	MGSSHHHHHHSSGLVPRGSHMAARELLQVGKDPYRGSGPVNSGPPCGSSKQSCHPSPKPGPNKPKKRCESSARQSDCGPN*
RISP-GFP-His	5’-ccccCCATGGCTGCTCGAGAATTACTG-3’	5’-ccccCCGCGGCCGCTTTGTATAGTTCATCCAT-3'	pet28a	MAARELLQVGKDPYRGSGPVNSGPPCGSSKQSCHPSPKPGPNKPKKRCESSARQSDCGPKDPGKGEELLTGVVPILVELDGDVNGHKFSVSGEGEGDATYGKLTLKFICTTGKLPVPWPTLVTTLTYGVQCFSRYPDHMKQHDFFKSAMPEGYVQERTIFFKDDGNYKTRAEVKFEGDTLVNRIELKGIDFKEDGNILGHKLEYNYNSHNVYIMADKQKNGIKVNFKIRHNIEDGSVQLADHYQQNTPIGDGPVLLPDNHYLSTQSALSKDPNEKRDHMVLLEFVTAAGITHGMDELYKAAALEHHHHHH*
RISP-mCherry	5'-CACCGAAGACACAATGAAAGCCTTTCTTGTGATC-3'	5'-CTACGAAGACGGCGAAccATTTGGGCCACAATCAGATTG-3'	pICH86988	MKAFLVIICILLATIVLSPSSTSAARELLQVGKDPYRGSGPVNSGPPCGSSKQSCHPSPKPGPNKPKKRCESSARQSDCGPNGSMVSKGEEDNMAIIKEFMRFKVHMEGSVNGHEFEIEGEGEGRPYEGTQTAKLKVTKGGPLPFAWDILSPQFMYGSKAYVKHPADIPDYLKLSFPEGFKWERVMNFEDGGVVTVTQDSSLQDGEFIYKVKLRGTNFPSDGPVMQKKTMGWEASSERMYPEDGALKGEIKQRLKLKDGGHYDAEVKTTYKAKKPVQLPGAYNVNIKLDITSHNEDYTIVEQYERAEGRHSTGGMDELYK*

Table S2. Secondary structure predictions from CD spectra

software	condition	α-helix	β-sheet	turn/ remainder	random coil
Selcon3	His-RISP	13.9	24	14.4	39.6
	His-RISP in TFE 50%	26.1	11.7	13.8	38
	His-RISP in TFE 80%	33.1	11.2	14.1	35.4
Contin	His-RISP	16	37	47	
	His-RISP in TFE 50%	26	27	47	
	His-RISP in TFE 80%	34	25	40	
